# Microbiota and gut neuropeptides: a dual action of antimicrobial activity and neuroimmune response

**DOI:** 10.1007/s00213-019-05224-0

**Published:** 2019-04-17

**Authors:** Julia Aresti Sanz, Sahar El Aidy

**Affiliations:** Department of Molecular Immunology and Microbiology, Groningen Biomolecular Sciences and Biotechnology Institute (GBB), University of Groningen, Nijenborgh 7, 9747 AG Groningen, The Netherlands

**Keywords:** Neuropeptides, Bacteria, Neuro-immune response, Gut-brain axis

## Abstract

The gut microbiota is comprised of a vast variety of microbes that colonize the gastrointestinal tract and exert crucial roles for the host health. These microorganisms, partially via their breakdown of dietary components, are able to modulate immune response, mood, and behavior, establishing a chemical dialogue in the microbiota–gut–brain interphase. Changes in the gut microbiota composition and functionality are associated with multiple diseases, in which altered levels of gut-associated neuropeptides are also detected. Gut neuropeptides are strong neuroimmune modulators; they mediate the communication between the gut microbiota and the host (including gut–brain axis) and have also recently been found to exert antimicrobial properties. This highlights the importance of understanding the interplay between gut neuropeptides and microbiota and their implications on host health. Here, we will discuss how gut neuropeptides help to maintain a balanced microbiota and we will point at the missing gaps that need to be further investigated in order to elucidate whether these molecules are related to neuropsychiatric disorders, which are often associated with gut dysbiosis and altered gut neuropeptide levels.

## Introduction

The gut microbiota comprises a complex community of metabolically active microorganisms that have a strong influence on a wide range of physiological processes, such as immune and nervous system development, food metabolism, cell growth and differentiation, or mood and behavior (Dinan and Cryan 2017; El Aidy et al. 2013; El Aidy et al. 2016; Erny et al. 2015; Rowland et al. 2018; Taylor and Holscher 2018). Though the composition of the gut microbiota is unique for each individual and therefore it is not possible to determine what defines a “healthy microbiota,” it seems clear that alterations in the composition of the gut microbiota, known as dysbiosis, are associated with multiple diseases, including neuropsychiatric disorders and inflammatory gastrointestinal diseases (Koopman and El Aidy 2017; Pusceddu et al. 2018). Thus, it is of great significance to investigate the mechanisms involved in the interplay between the microbiota and the host.

One of the ways by which the gut bacteria establish their intimate relationship with their host is through the production of biologically active molecules. Bacteria produce these molecules by breaking down dietary compounds that reach the intestine and/or the indigenously produced compounds that are expelled from the intestine (Dodd et al. 2017; Strandwitz 2018; Zhang and Davies 2016). Products of bacterial breakdown range from immunomodulatory to antimicrobial and neuroactive compounds that not only have a local action on the host but can also reach the blood stream to have an impact on distant parts of the body (Lyte and Cryan 2014).

Neuropeptides are now in the spotlight as one of the potential mediators of the exchange of information between gut bacteria and other tissues and organs. Their role as modulators of neuronal and immune functions is well known and reveals a strikingly complex network through which neuropeptides exert multiple functions. Although the term “neuropeptide” is commonly used in the context of the central nervous system (CNS), the enteric nervous system (ENS) is another major source of production of these peptides. Therefore, in this article, we will use the term “gut neuropeptides” to specifically refer to neuropeptides which are produced in the ENS, as opposed to other gut peptides which are also produced in the intestinal epithelium. This term also reinforces the idea that gut neuropeptides are able to exert an extraintestinal action by signaling to distant organs, such as the brain. Interestingly, several gut neuropeptides also have antimicrobial activity; for instance, neuropeptide Y (NPY) and substance P (SP), which have been shown to inhibit the growth of *Escherichia coli* (Hansen et al. 2006). However, the antimicrobial activity of gut neuropeptides has not been studied in detail yet. Considering that gut antimicrobial neuropeptides are produced not only in the CNS but also in the ENS and their respective receptors are widely expressed along the gastrointestinal tract (GIT), it is important to unravel the function of these peptides in the context of gut microbiota homeostasis, which is key for a healthy state of the host. Finally, gut neuropeptides might also be key regulators of the so-called microbiota–gut–brain axis; for example, through their receptors expressed on the vagus nerve, the main communication route between the gut and the CNS (Holzer and Farzi 2014). In this review article, we will describe the different types of antimicrobial peptides (AMPs) that are present in the gut and their mode of action. Thereafter, we will focus on gut neuropeptides and will address their direct and indirect function in maintaining homeostasis.

## Antimicrobial peptides in the gastrointestinal tract

The GIT is a major entry point of microbes, and so, the organ has developed a complex defense mechanism to protect the host from diseases, as part of the innate immune system. One of the components of this defense barrier is the AMPs. In the GIT, AMPs are produced not only by the intestinal epithelium but also by the gut microbiota in the lumen (Table 1). As presented in Table 1, human defense peptides have a rather broad spectrum of action, whereas their bacterial counterparts display a much narrower spectrum. This is due to a very specific mode of action of bacterial AMPs where the antimicrobial action takes place upon high-affinity binding to receptors in the cell envelope (Martinez et al. 2016).

**Table 1:** Antimicrobial peptides in the gastrointestinal tract. Summary of host-derived and microbiota-derived antimicrobial peptides including producing cell types and antimicrobial spectrum

Source	AMP family	Cell type	Antimicrobial activity	References
Microbiota	Lantibiotics	Gram-positive bacteria	*S. pneumoniae*, *S. aureus*, *S. pneumoniae*, *C. difficile*, *E. faecium*	(Dawson 2007)
	Microcins	Gram-negative bacteria	*E. coli*, *S. enterica*, *E. cloacae*, *K. pneumoniae*, *Citrobacter*, *Shigella*	(Duquesne et al. 2007)
Host	Defensins	Paneth cells, monocytes, macrophages, T and B cells, dendritic cells	*S. aureus*, *P. aeruginosa*, *E. coli*, *L. monocytogenes*, *S. typhimurium*, *C. difficile*, *C. albicans*, *B. fragilis*, *E. faecalis*, *S. pyogenes*, *E. faecium*, *S. cerevisiae*, *S. pneumonia*, *B. cepacia*, HPV, HIV, influenza virus	(Sivieri et al. 2017)
	Phospholipase A2	Paneth cells	*E. coli*, *S. typhimurium*, *L. monocytogenes*, *S. aureus*, *B. anthracis*, *P. aeruginosa*, *B. subtilis*	(Timo J. Nevalainen et al. 2008)
	Cathelicidins	Enterocytes, macrophages, epithelial cells	*E. coli*, *K. pneumonia*, *P. aeruginosa*, *N. gonorrhoeae*, *Streptococcus sp.*, *H. pylori*, *Shigella sp.*, *Salmonella sp.*, *C. albicans*	(Sivieri et al. 2017)
	RegIII lectins	Enterocytes, enteroendocrine cells	*L. monocytogenes*, *Y. pseudotuberculosis*, *C. rodentium*, *S. enteritidis*, *S. enterica*, *Enterococcus*, *C. butyricum*, *L. reuteri*, *E. coli*, *Bacteroides spp.*	(Miki et al. 2017)
Host neuropeptides	NPY	Enteric neurons, neutrophils, monocytes, macrophages, fibroblasts	*E. coli*, *E. faecalis*, *Candida spp.*, *P. aeruginosa*, *S. mutans*, *L. acidophilus*, *L. major*, *M. catarrhalis*, *H. influenza*, *A. caviae*, *A. actinomycetemcomitans*	(Augustyniak et al. 2012)
	SP	Enteric neurons, neutrophils, monocytes, macrophages, lymphocytes B, lymphocytes T, dendritic cells, natural killers, mast cells, fibroblasts	*E. coli*, *C. albicans*, *P. aeruginosa*, *S. mutans*, *L. acidophilus*, *K. pneumoniae*, *E. faecalis*, *P. vulgaris*, *M. catarrhalis*, *H. influenza*, *S. aureus*, *A. actinomycetemcomitans*	(Augustyniak et al. 2012)
	α-MSH	Enteric neurons, neutrophils, monocytes, macrophages, lymphocytes B, lymphocytes T, dendritic cells, natural killers, mast cells	*Escherichia coli*, *Staphylococcus aureus*, *Candida albicans*, *Trypanosoma brucei*	(Augustyniak et al. 2012)
	CGRP	Enteric neurons, macrophages, lymphocytes T, dendritic cells	*E. coli*, *C. albicans*, *P. aeruginosa*, *S. aureus*, *E. faecalis*, *S. mutans*, *L. acidophilus*, *M. catarrhalis*, *H. influenzae*	(Augustyniak et al. 2012)
	AM	Enteric neurons, neutrophils, macrophages, mast cells	*B. fragilis*, *E. coli*	(Allaker et al. 1999; Augustyniak et al. 2012)
	VIP	Enteric neurons, monocytes, macrophages, dendritic cells, T lymphocytes, B lymphocytes, mast cells	*T. brucei*	(Augustyniak et al. 2012; Delgado et al. 2009)

### Antimicrobial peptides produced by the gut microbiota

Gut bacteria represent a major source of AMPs production in the GIT. These bacteria synthesize the so-called bacteriocins. Similar to AMPs produced by human cells, bacteriocins are small, cationic peptides that can easily interact with bacterial membranes. So far, 177 bacteriocins have been identified and sequenced; 88% of them are produced by Gram-positive bacteria, whereas the remaining 12% are produced by Gram-negative bacteria and Archaea. It is also remarkable that most of the reported Gram-positive bacteriocins producers belong to the group of lactic acid bacteria, which carry out fermentation of sugar into lactic acid (http://bactibase.hammamilab.org/statistics.php). However, it cannot be concluded that lactic acid bacteria are the only bacteriocin producers; instead, given the interest that they pose for the food industry, it is likely that most of the research has been focused on this group of bacteria and accordingly more bacteriocins have been reported to be produced by lactic acid bacteria.

Bacteriocins are classified into many different subgroups due to the heterogenicity of this group of molecules. Bacteriocins that are produced by Gram-negative bacteria are referred to as microcins, small peptides, or colicins, which are larger proteins. Microcins are subsequently divided into class I (< 5 kDa, containing post-translational modifications) and class II (5–10 kDa, without post-translational modifications) (Hassan et al. 2012). Despite their structural diversity, microcins share an interesting mechanism of action that has been named the “Trojan Horse” strategy. Some microcins such as MccJ25 and MccE492 mimic the structure of essential bacterial molecules and take advantage of the natural receptors for these ligands, which allow them to enter and kill the target bacteria. On the other hand, MccC7 and MccC59 are secreted as harmless molecules and further transformed into toxic derivatives once they enter the susceptible bacteria (Duquesne et al. 2007). An interesting example of how microcins are relevant for the host was shown recently using a mouse model of intestinal inflammation. In the study of Sassone-Corsi et al., probiotic *E. coli* Nissle 1917, which produces microcins, was able to restrict the expansion of other competing Enterobacteriaceae during inflammation, including pathogenic *E. coli* and *Salmonella enterica*. Also, therapeutic administration of *E. coli* Nissle was sufficient to effectively displace enteric pathogens from their niche due to their production of microcins (Sassone-Corsi et al. 2016). The second subgroup of Gram-negative bacteriocins, colicins, are larger peptides produced by some strains of *E. coli* and other related Enterobacteriaceae. They are active against *E. coli* and closely related bacteria such as *Salmonella* (Braun et al. 1994). Similarly to microcins, colicins are quite a diverse group that includes up to 30 types of proteins which slightly differ in lethal activity and mode of action (Smarda and Smajs 1998). The mechanism by which colicins kill bacteria has been extensively studied and consists of three main steps. Initially, colicins bind to the outer membrane proteins of the target cell through the receptor binding domain. Then, the translocation domain located in the N-terminus of the protein allows colicins to enter the target cell: depending on which protein complex they interact with, colicins are subdivided into Tol-dependent or Ton-dependent colicins. These periplasmic protein complexes allow colicins to translocate and reach the inner membrane where the killing activity takes place by different mechanisms: pore formation, DNAse activity, or inhibition of protein synthesis (Cascales et al. 2007). Interestingly, a very recent study analyzed clinical isolates of *E. coli* from the intestinal mucosa of patients suffering from inflammatory bowel disease (IBD) and found a higher frequency of bacteriocinogeny when compared to healthy subjects. The study found that IBD *E. coli* strains had a higher prevalence of virulence determinants when compared to healthy controls, suggesting that IBD *E. coli* strains closely resemble their pathogenic counterparts. One of these determinants was group B colicins, which are actually encoded in plasmids containing additional virulence genes. This association between IBD and group B colicins could therefore reflect additional virulence determinants present in the colicin plasmids highly prevalent in IBD (Micenková et al. 2018). The use of recombinant colicins directed to specific pathogenic *E. coli* strains present in IBD has been proposed as a therapeutic method for IBDs (Kotłowski 2016). Thus, genetically modified *E. coli* Nissle 1917 could be used as a vehicle to introduce specific colicins in the GIT that would reinforce the antimicrobial action that this strain exerts through the production of microcins (Kotłowski 2016). However, it is important to note that this hypothesis has not been yet tested in the laboratory, so in vitro and in vivo experiments are needed to confirm the potential of colicins as therapeutic agents in intestinal inflammation.

Bacteriocins produced by Gram-positive bacteria are divided into lantibiotics and non-lantibiotics. Lantibiotics constitute the Gram-positive counterpart of microcins; they are small peptides, below 5 kDa, and contain unusual post-translationally modified residues such as lanthionine and 3-methly-lathionine (Perez et al. 2014). The first lantibiotic ever discovered was Nisin A (Rogers and Whittier 1928), and a lot of studies are still being done on how to genetically modify this peptide to create derivatives improving its antimicrobial properties (Field et al. 2012; Li et al. 2018). Lantibiotics are quite unique molecules; they present unusual thioether-containing amino acid residues that form ring structures within the peptides (Jack and Jung 2000), which is probably linked to their complex mechanism of action. The mode of action of these antimicrobials was for a long time thought to be restricted to the pore formation mechanism of the prototypic lantibiotic nisin (Garcerá et al. 1993). However, upon structural derivatization, new insights reveal a complex set of modes of action that are combined to eventually disrupt bacterial growth. For instance, nisin takes the pore formation process one step further, increasing the specificity and stability of the pore through the interaction with lipid II. Lipid II is a molecule that is anchored to cell membranes and acts as a precursor in the synthesis of peptidoglycan by translocating across the membrane the building blocks required for its synthesis (van Heijenoort 2007). By binding to lipid II, nisin is able to greatly improve the pore-forming efficiency (Breukink et al. 1999) and this mechanism has also been described in other lantibiotics, such as the two-peptide lantibiotic lacticin 3147 (Wiedemann et al. 2006) and subtilin (Parisot et al. 2008). Interestingly, the interaction with lipid II has another effect that also increases the potency of these molecules as antibiotics. As lipid II is a precursor in the synthesis of peptidoglycan, the interaction with nisin sequesters lipid II from its normal function and results in the inhibition of cell membrane biosynthesis, which creates the dual effect of this lantibiotic (Wiedemann et al. 2001).

In a similar way to microcins, Gram-positive bacteriocins also have great therapeutic potential. For example, enterococcal bacteriocin 21 might constitute an effective approach to decolonize antibiotic-resistant enterococci from the gut. In a recent study, commensal *Enterococcus faecalis* delivering bacteriocin 21 was used to specifically disrupt the growth of multidrug-resistant enterococci during infection in the mammalian GIT. Thus, a particular niche from the gut microbiota was inhibited without altering the rest of the microbial population, which poses a useful strategy to treat infections caused by antibiotic resistant strains that might otherwise be very challenging to cure (Kommineni et al. 2015).

### Antimicrobial peptides produced by the intestinal mucosa

The intestinal epithelium provides the first line of defense against microorganisms that reach the gut lumen and can be pathogenic for the host. Intestinal epithelial cells are mainly enterocytes, but they also comprise enteric neurons, enteroendocrine cells, tuft cells, and secretory cells, which include goblet and Paneth cells. Goblet cells produce mucin and Paneth cells secrete AMPs, creating a physical and biochemical barrier that ensures the proper segregation between host and microorganisms, helping to avoid infections (Allaire et al. 2018). It must be noted, however, that Paneth and goblet cells are not the only source of host-derived AMPs. These epithelial secretory cells are aided by classic immune cells located in the lamina propria, which include lymphocytes, macrophages, and dendritic cells for the production of AMPs (Charles et al. 2001).

So far, five different AMPs and proteins have been found to be produced by Paneth cells: defensins, cathelicidins, lysozyme C, phospholipase A2, and REGIIIα/β/γ. Lysozyme C, a glycoside hydrolase that cleaves specific residues of peptidoglycan causing lysis of the bacterial membrane, was firstly found to be expressed in Paneth cells (Mason and Taylor 1975). Phospholipase A2 was also found to be synthesized in Paneth cells (Nevalainen et al. 1995) and described as a bactericidal agent against *E. coli* and *Listeria monocytogenes*. Furthermore, Paneth cells produce REGIIIα, a C-type lectin also known as HIP/PAP (hepatocarcinoma–intestine–pancreas/pancreatic-associated protein), which is produced in enteroendocrine cells as well (Lasserre et al. 1999). REGIIIα, as many other antimicrobial peptides, is able to kill bacteria by formation of a permeabilizing transmembrane pore, although this mechanism is inhibited by lipopolysaccharides (LPS), which is why this peptide is only effective against Gram positive-bacteria (Mukherjee et al. 2013). Additionally, REGIII peptides play an important role during the initial establishment of the gut microbiota in germ-free mice. Indeed, REGIIIγ and REGIIIβ expressions have been shown to peak during the process of gut bacterial colonization in both the small intestine and the colon of mouse, coinciding with induced expression of innate immune molecules phospholipase A2 (Pla2g2a) and resistin-like beta (Retnlβ) in the colon (El Aidy et al. 2012). Later on, the expression of REGIII peptides in the colon returned to basal levels detected in germ free animals. This suggests that REGIII peptides exert an antimicrobial function in the small intestine, while Pla2g2a and Retnlβ exert this antimicrobial function in the colon (El Aidy et al. 2012).

The major AMP components in the intestinal mucosa are defensins. So far, ten defensins have been identified and divided in two groups according to structural features: human α-defensins (HDs) and human β-defensins (HBDs). Defensins are small, cationic peptides with a rather broad spectrum of antimicrobial activity in vitro and they exert their function by creating micropores in the bacterial membranes that cause the leak of the cell’s content, loss of structure, and eventual cell death (Cobo and Chadee 2013; Cunliffe 2003). They are synthesized as propeptides and are subsequently cleaved by proteases to form the mature, active forms of the peptides (Valore & Valore and Ganz 1992). HDs are produced by Paneth cells and are therefore in charge of protection in the small intestine, whereas HBDs play the same role in the colon, where they are secreted by epithelial cells. Additionally, HDs are produced by immune cells including monocytes, macrophages, T and B cells, which also produce HBDs together with dendritic cells (Dutta and Das 2016).

The other family of AMPs produced in the GIT by the host is cathelicidins. Among cathelicidins, only LL-37 is expressed in humans. Cathelicidins and defensins share some common features; LL-37 is also synthesized as a propeptide, which is later cleaved by a protease to release the mature peptide with antimicrobial activity (Zaiou and Gallo 2002). Furthermore, the mode of action of LL-37 is also based on bacterial membrane disruption by pore formation (Xhindoli et al. 2016). However, in contrast to defensins, which are only expressed in the small intestine, LL-37 expression in this region of the intestinal tract is rather low, while it is highly abundant in the colon (Schauber et al. 2003), where it is secreted by colonocytes (Hase et al. 2002).

### Antimicrobial gut neuropeptides

There is increasing evidence that gut neuropeptides are one of the axes of communication between the gut microbiota and the host (El Aidy et al. 2016) and they might also be playing a role as antimicrobial agents. Gut neuropeptides are structurally similar to regular antimicrobial peptides; they are small (< 10 kDa), cationic, and amphipathic molecules and also share similarities with other AMPs in their mode of action. Even though their antimicrobial properties have not yet been studied in detail, there are multiple indications that gut neuropeptides such as NPY, SP, α-melanocyte stimulating hormone (α-MSH), vasoactive intestinal peptide (VIP) calcitonin gene-related peptide (CGRP), and adrenomedullin (AM) might be having an important role in the regulation of the gut microbiota composition.

**Fig. 1:**
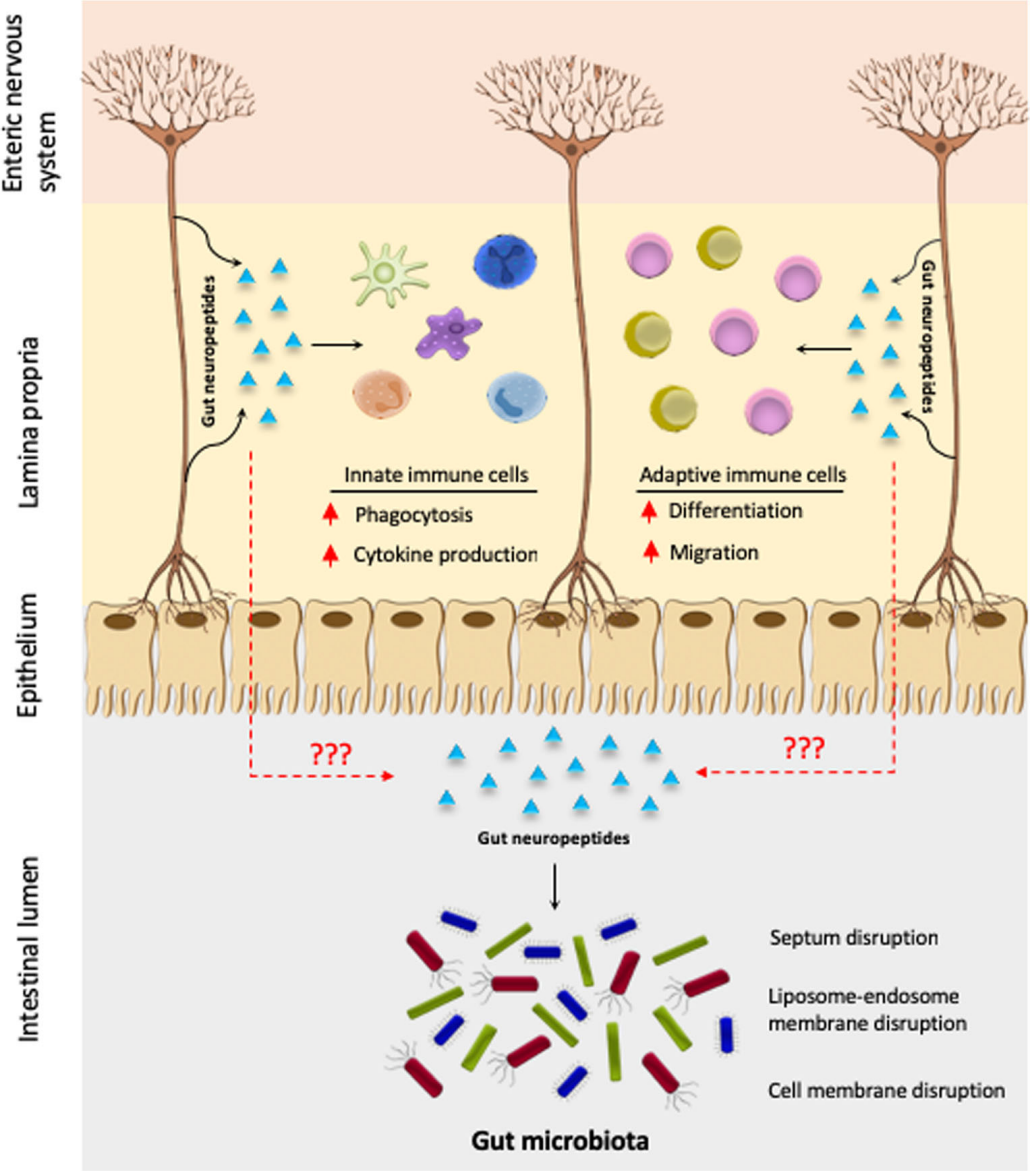
Direct and indirect effects of gut neuropeptides in the GIT. Upon sensing of stressful stimuli, enteric neurons release gut neuropeptides that induce a response in innate and adaptive immune cells, which also secrete these peptides, resulting in a strong response to bacterial imbalance. Additionally, if gut neuropeptides cross the epithelial barrier, they could exert a direct antimicrobial activity in the intestinal lumen by different killing mechanisms

Gut neuropeptides emerge not only from enteric neurons in response to different stimuli (Fig. 1) but also from immune cells and enteroendocrine cells (Yoo and Mazmanian 2017). The ENS extends from the myenteric plexus of the intestine and reaches the intestinal epithelium, being able to sense different stimuli across several layer of the intestine and to regulate multiple intestinal functions (Furness 2012). For instance, sensory neurons reaching the epithelium can detect stressful stimuli such as pathogenic bacteria and secrete SP, which subsequently induces cytokine secretion from immune cells (Fig. 1). Gut neuropeptides, including gut antimicrobial neuropeptides, are therefore part of an extremely complex network between the nervous and immune systems where they are playing a key modulatory role.

NPY is a 36 amino acid peptide produced by enteric neurons, and it regulates a wide variety of physiological processes in the gut such as gut motility, inflammation, cytokine secretion, and epithelial permeability (Chandrasekharan et al. 2013). In the gut, Y1, Y2, Y4, and Y5 receptors (which belong to the family of G protein coupled receptors, GPCRs) present in enteric neurons and enterocytes mediate the gastrointestinal motility control of NPY (Holzer et al. 2012). Furthermore, sufficient evidence supports the role of NPY as a key modulator of the neuroimmune crosstalk (Bedoui et al. 2003). Interestingly, NPY has been reported as an antimicrobial agent in vitro against *Cryptococcus neoformans*, *Candida albicans*, and *Arthroderma simii*, with a minimal inhibitory concentration of 7 μM (Vouldoukis et al. 1996). SP is a much smaller peptide of only 11 amino acids, highly conserved and expressed in enteric nerves, enteric sensory neurons, and enteric immune cells. As NPY, it has multiple roles in gut physiology such as mediating inflammation, nociception, muscle contraction, and gut motility, as well as being another modulator of the neuroimmune communication, via neurokinin receptors NK1R and NK2R, which are also GPCRs (Koon and Pothoulakis 2006). SP has also been shown to display antimicrobial activity in vitro against *Staphylococcus aureus*, *E. coli*, *E. faecalis*, *Proteus vulgaris*, *Pseudomonas aeruginosa*, and *C. albicans*. The antimicrobial activity of SP and NPY against *E. coli*, *E. faecalis*, *P. aeruginosa*, and *C. albicans* was confirmed in a different study, which also showed activity of both gut neuropeptides against *Streptococcus mutans* and *Lactobacillus acidophilus* (El Karim et al. 2008). However, two more recent studies failed to show inhibition of growth of *S. aureus* and another *Pseudomonas* strain (*P. fluorescens*) upon exposure to SP (Mijouin et al. 2013; N’Diaye et al. 2016). Furthermore, in another study, both SP and NPY only showed antimicrobial activity against *E. coli*, failing to have an effect on *S. aureus* and *C. albicans* (Hansen et al. 2006). Resistance of *S. aureus* to SP and NPY was also shown by Karim et al., so it seems like this bacterium is not affected by any of these gut neuropeptides. The discrepancies in the results obtained with respect to certain strains might be due to the use of laboratory strains versus fresh isolates or to the use of different methods, but given their structural features, their production in abundance in the gut and the physiological roles of these two peptides, their antimicrobial capacity needs to be further investigated.

Another important gut neuropeptide is α-MSH, a 13 amino acid peptide that emerges from the post-translational processing of POMC (proopiomelanocortin). As other gut neuropeptides, it is an important neuroimmune modulator being a potent antiinflammatory molecule, which allows it to regulate intestinal permeability (Váradi et al. 2017). α-MSH also exerts its function through another member of the GPCR family of receptors; melanocortin receptors (MCRs) bind α-MSH triggering different signaling pathways that regulate a variety of functions. In the gut, MC1R and MC3R induction by α-MSH modulates its antiinflammatory response through cyclic adenosine 3′,5′-monophosphate (cAMP) signaling (Singh and Mukhopadhyay 2014). α-MSH antimicrobial activity was first shown against *E. coli*, *C. albicans*, and *S. aureus* (Cutuli et al. 2000), which was later extended to *Cryptococcus neoformans* (Masman et al. 2006) and *C. vaginitis* (Catania et al. 2005).VIP is another gut neuropeptide which has been shown to display promising antimicrobial activity, at least against some pathogens (El Karim et al. 2008). VIP is a 28 amino acid peptide that is also present in the GIT, where it regulates different function through the VPAC2 receptors, which belong to GPCR family too. Apart from being an important immune regulator, VIP regulates vasodilatation in the gut, as well as motility (Mario Delgado and Ganea 2013). Regarding its antimicrobial activity, VIP was shown to be effective in killing *S. mutans*, *E. coli*, *P. aeruginosa*, and *C. albicans* in vitro. Additionally, VIP was also able to kill the pathogen *Trypanosoma brucei* in another in vitro study (M. Delgado et al. 2009). However, not many follow-up studies on the antimicrobial properties of VIP are available. Instead, a few studies with synthetic analogues of the peptide with improved stability have shown enhanced antimicrobial activity of VIP. Indeed, modified VIP peptides were able to kill *S. mutans*, *Micrococcus luteus*, and pathogenic *E. faecalis*, while the native peptide was ineffective against these bacteria against *S. aureus* and *E. coli* compared to the native form of the peptide (Campos-Salinas et al. 2014). Using also analogues of VIP, Xu et al. were able to demonstrate antimicrobial activity against *S. aureus* and *E. coli* that was increased when compared to the natural form of the peptide (Xu et al. 2017). Finally, CGRP is another gut neuropeptide that has been investigated as an antimicrobial peptide. In its mature form, it is composed of 37 amino acids and has two splicing variants; α-CGRP and β-CGRP. α-CGRP is predominantly expressed not only in the CNS but also in the peripheral nervous system, whereas β-CGRP is mostly found in the ENS. This peptide causes vasodilation in the GIT and has a protective role against ischemia (Ma 2016). Regarding its antimicrobial activity, CGRP is able to affect the growth of *E. coli*, *C. albicans* and *P. aeruginosa* (El Karim et al. 2008), therefore having quite a restricted spectrum of action, as compared with the other gut neuropeptides discussed before. Within the CGRP family of peptides, it is also important to mention AM. This 52 amino acid peptide is generated by post-translational enzymatic processing of preproadrenomedullin, which also results in the formation of its gene-related peptide proadrenomedullin N-terminal peptide (PAMP) (Bełtowski and Jamroz 2004). AM shares structural and functional similarities with CGRP, such as the presence of an internal molecular ring and a central helical region for receptor binding, and both peptides exert their functions through the calcitonin receptor-like receptor (CRLR) (Hay and Walker 2017; Pérez-Castells et al. 2012). AM and PAMP are found across the entire GIT, being specially abundant in neuroendocrine cells, and they regulate growth of the intestinal epithelium, water, and ion transport in the colon, intestinal motility, and vasodilatation (Martínez-Herrero and Martínez 2016). Antimicrobial properties have also been described for both peptides; antimicrobial activity of AM was initially shown against a range of bacteria known to be members of the microbiota of different mucosal surfaces, including intestinal mucosa. AM was able to inhibit the growth of *Bacteroides fragilis* and *E. coli* (Allaker et al. 1999), and this was extended in a later study to PAMP, which was actually shown to be even more potent than AM against *E. coli* (Marutsuka et al. 2001).

As shown in Table 1, the antimicrobial activity of gut neuropeptides has mainly been tested against pathogenic bacteria. Although of high relevance, it is still necessary to study antimicrobial activity of gut neuropeptides on commensal bacteria. Not only inflammation can be caused by colonization of pathogens, but also certain strains of commensal bacteria, such as *E. coli*, can trigger the production of cytokines and induce an inflammatory state in the gut that can translate to the brain. (Kittana et al. 2018). Therefore, the role of these peptides as antimicrobial agents against commensal strains should be further explored in order to understand their potential impact in the host health.

## Modes of action of antimicrobial gut neuropeptides

As mentioned before, the common structural features of host defense peptides and antimicrobial gut neuropeptides allow the latter to exert their function through the same mechanisms of action on the target microorganisms. These include membrane disruption, interference with cell division, and metabolism or disruption of ATP synthesis among others. Furthermore, gut neuropeptides have an additional capacity of interacting with the neuro- and immune systems, which ultimately causes the release of other molecules with antimicrobial activity. This combined action raises a great potential for gut neuropeptides to be used with therapeutic purposes in order to treat diseases associated with microbiota alterations, especially in light of the increasing resistance to conventional antibiotics that is putting at risk the use of these drugs.

### Direct antimicrobial effects of gut neuropeptides

The mechanism of action of antimicrobial peptides has been studied in depth mostly in host defense peptides, but the structural similarities of gut neuropeptides, which are also small, cationic molecules, allow them to exert their function in a similar way. The predominant antimicrobial mode of action of gut neuropeptides consists of binding and disruption of the bacterial cell wall. Indeed, membrane disruptive mechanisms have been shown to drive the action of NPY (Thomas et al. 2005), α-MSH (Madhuri et al. 2009), and AM (Allaker et al. 2006), all of which contain a rich combination of positively charge amino acids and hydrophobic residues in their C-terminal fragment. However, non-disruptive mechanisms also exist and will be discussed later on.

Membrane-disruptive peptides usually form α-helical structures with accumulation of positively charged residues towards the C-terminal side of the peptide (Powers and Hancock 2003). This allows the initial step to approach their target, which occurs through electrostatic interactions of such residues with the anionic LPS that coat the outer bacterial membrane. By displacement of Mg^2+^ and Ca^2+^ ions that are usually interacting with LPS, antimicrobial peptides create local disturbances in the membrane, allowing their translocation and making the inner cytoplasmic membrane accessible (Hancock and Chapple 1999). The next step consists of the reorientation of the peptide to interact with the inner membrane. Different mechanistic models have been proposed for the interaction of these peptides with inner bacterial membranes, and although, it is not yet clear which is correct, they all lead to the disruption and depolarization of the membrane, which rapidly causes cell death (Sato and Feix 2006). It is easy to speculate that there might not be one unique model to explain the mode of action of these peptides, but instead, they might all be correct depending on the physicochemical properties of each antimicrobial peptide.

Interestingly, another rather non-traditional mode of action has been described to take place in the trypanolytic activity of gut neuropeptides AM and VIP. Trypanosomiasis is known to cause intestinal damage in humans, as an inflammatory state is induced upon the invasion of this parasite with increased cytokine levels and intestinal permeability (Ben-Rashed et al. 2003). Studying the antimicrobial activity of different gut neuropeptides on *T. brucei*, an unusual antimicrobial mechanism was found in which the peptides are subjected to endocytosis through the flagellar pocket of this microorganism. Next, trafficking of the peptides through the endosomal network allows them to reach the lysosomes, where they disrupt endosome–lysosome vesicles. Disruption of these membranes causes the release of the gut neuropeptides to the cytosol, together with glycolytic enzymes, which leads to a metabolic failure and finally to cell death (Delgado et al. 2009).

Finally, AM has also been shown to have a distinct, unusual mechanism of action against *S. aureus*. Staphylococci division occurs though the inwards growth of the peripheral cell wall and formation of a transverse cross wall, known as septum. Then, the newly synthesized peptidoglycan undergoes localized hydrolysis that results in complete cell separation (Giesbrecht et al. 1998). In *S. aureus*, AM has been shown to disrupt the formation of the septum (Allaker et al. 2006), which is known to lead to cell death in other organisms.

Even though these results are promising and present gut neuropeptides as potential modulators of gut microbiota homeostasis by direct antimicrobial activity, some questions need to be addressed before they can be implemented as therapeutic agents. The studies discussed above are in vitro studies, and it is not yet clear whether these gut neuropeptides reach the gut microbiota in sufficient amounts. For them to be effective in direct killing of bacteria, gut neuropeptides should be able to reach the gut lumen or areas in close proximity to the intestinal epithelium, where they may exert a very important role in gut microbiota homeostasis. Another concern for their potential therapeutic implementation is that, as mentioned before, most of the studies so far have tested antimicrobial activity against pathogenic bacteria, which leaves commensal bacteria unexplored. It would therefore be crucial to unravel whether these peptides can keep infections under control without altering the composition of commensal bacteria residing in the GIT. In light of the growing resistance of pathogens to the currently used broad-spectrum antibiotics, exploring the therapeutic possibilities of gut neuropeptides is extremely significant; these peptides could be active against resistant strains and their seemingly restricted spectrum of action would not influence other untargeted bacteria, reducing the possibilities of developing resistance.

### Indirect antimicrobial effects of gut neuropeptides

Besides their antimicrobial action on microbes, gut neuropeptides have much more sophisticated roles and probably the more important antiinfective functions of these molecules rely on their neuroimmune modulatory properties. Such features have allowed the host to develop a complex and intrinsic network of defense mechanisms to protect itself from microbial invasion. Gut neuropeptides respond to gut microbiota alterations by mediating the so called neurogenic inflammation (Houser and Tansey 2017). At the same time, neuroimmune modulation by gut neuropeptides translates to changes in the ENS, which can eventually reach the brain and impact mood and behavior. Thus, inflammation in the GIT results in changes in ENS, such as increased number of enteric neurons, altered levels of gut neuropeptides and changes in the motor circuits of the intestine (Margolis and Gershon 2016).

Nociceptors, which are specialized peripheral sensory neurons, respond to damaging stimuli in the GIT such as infection by releasing gut neuropeptides. SP, CGRP, AM, and NPY are released from these neurons and mediate neurogenic inflammation as a defense mechanism for the host. This way, during the effector phase of inflammation, sensory neurons produce neuropeptides that promote proliferation and migration of immune cells, guiding them to the specific site of damage (Chiu et al. 2012). NPY, SP, and CGRP are able to stimulate the release of proinflammatory cytokines, chemokines, and arachidonic acid from immune and non-immune cells, while VIP, α-MSH, and AM seem to have the opposite effect. Altogether, they mediate neurogenic inflammation (Fig. 1) (Mitchell and King 2010; Souza-Moreira et al. 2011). A detailed description of the action of neuropeptides on the immune system is provided in the review articles by Souza-Moreira et al. and Mitchell et al. Interestingly, one of the properties that make gut neuropeptides such potent mediators of the inflammatory response is their often synergetic activity with other molecules, which allows them to orchestrate an intense and efficient response. For instance, SP and CGRP have been shown to act jointly on peripheral blood mononuclear cells (PBMC). This synergetic effect is in line with the additive effects that these peptides are known to have in other physiological processes involved in the inflammatory response; vasodilatation, vascular permeability, and hyperalgesia (Cuesta et al. 2002). SP has been shown to enhance the response of macrophages and monocytes by being able to strongly increase the release of cytokines and chemokines from these cells in response to LPS (Sipka et al. 2010). Another example of this coordinated action with other molecules is VIP, which is able to facilitate the bactericidal activity of human cathelicidin LL-37; when used in combination, VIP and LL-37 can keep their antimicrobial activity even at physiological concentrations of NaCl, at least against *E. coli* and *P. aeruginosa* (Ohta et al. 2011).

The complex picture of gut neuropeptide modulation of the immune response through their capacity to regulate innate and adaptive immune response via different receptors gives rise to a great variety of modes of action. For example, activation of neutrophil receptor Y5 by NPY potentiates a respiratory burst resulting in an increase of reactive oxygen species (ROS), one of the critical functions of neutrophils. Conversely, when NPY binds to Y1 and Y2 receptors, phagocytosis of *E. coli* is inhibited. Furthermore, the effect of this gut neuropeptide on neutrophils is also concentration-dependent; at low doses, NPY promotes phagocytosis, whereas at higher doses phagocytosis is inhibited and the respiratory burst is potentiated to eliminate the pathogen (Bedoui et al. 2008). On the other hand, a recent study using a SP agonist showed that stimulation of NK1R of dendritic cells promotes the maturation of these cells together with decreased secretion of IL-10 (Janelsins et al. 2013). Moreover, in line with its antiinflammatory effect described before, α-MSH drives the maturation of macrophages by interaction with MC1R receptors in these cells. Interestingly, this process has been specifically shown to kill *C. albicans* in a rat *vaginitis* model, again through cAMP signaling (Ji et al. 2013). CGRP is also able to regulate cells of the innate immune system. For instance, binding of the peptide to CRLR of macrophages induces IL-6 and TNF-α (Fernandez et al. 2001).

Regarding the interaction of gut neuropeptides with adaptive immune cells, SP has been reported to induce maturation of human memory CD4+ T cells into Th17 cells upon induction of IL-1β production in monocytes, an effect that occurs upon induction of NK1R in these cells (Cunin et al. 2011). α-MSH is also able to upregulate cytokine IL-10 in dendritic cells via MC1R, leading to the induction of regulatory T cells and eventual inhibition of effector T cells which points to an immunosuppressive phenotype (Auriemma et al. 2012). Another example is the ability of NPY to induce chemotaxis, adhesion to epithelial cells, and transepithelial migration of dendritic cells through Y1 receptor activation (Buttari et al. 2014).

In summary, gut neuropeptides allow the host to have a strong response to bacterial infections; damaging stimuli are sensed by sensory neurons, which in turn secrete these peptides that can directly act on microbes and at the same time induce inflammation through the interaction with immune cells in the intestinal mucosa. However, this complicated response has its implications on the GIT as it involves neuropeptide-mediated gastrointestinal inflammation on the ENS. An inflammatory state of the bowel induces severe changes in the ENS that result in gastrointestinal dysfunction. It has been shown that inflammation causes multiple changes in the intrinsic circuitry of the ENS, such as neuronal hyperexcitability, increased synaptic facilitation, and decreased inhibitory neuromuscular transmission (Krauter et al. 2007; Linden et al. 2004; Strong et al. 2010). This profound neuronal alteration ultimately leads to major, long-lasting disruption of the intestinal motor activity. Interestingly, bowel dysfunction is observed not only at the site of inflammation but also in non-inflamed regions of the gut, which might be explained by the long lasting alteration of enteric neuronal circuits. Altered motility and secretion can be found at distant, non-inflamed regions of the GIT, as shown in an experimental model of colitis in guinea pigs (Hons et al. 2009). In this study, non-cholinergic secretion was found to be decreased in the ileum where no inflammation was observed, and this change was associated with a decrease in excitatory synaptic transmission in secretomotor neurons. In contrast, ileal cholinergic neurons were more excitable, while action potentials in primary afferent neurons were broader than in a normal, non-inflamed state (Hons et al. 2009). Although the mechanism behind the distant effect of inflammation on non-affected regions of the gut is not yet understood, it seems clear that altered enteric neuronal function caused by neuropeptide-mediated inflammation can have effects in other regions of the GIT and this might be explained by a neuronal-mediated spread of the inflammation, resulting in profound and long lasting changes in the ENS across the whole bowel.

## Conclusion

The gut microbiota is emerging lately as a key regulator of host health and disease. However, the mechanisms employed by these microbes to communicate with distant organs such as the brain are only beginning to be understood and antimicrobial gut neuropeptides are likely be involved in this process. Gut neuropeptides have the capacity to regulate gut microbiota homeostasis through both direct antimicrobial effects and neurogenic inflammation and therefore should be considered as potential therapeutic targets for diseases where this function is affected. Gut neuropeptides do seem to have a rather narrow spectrum as antimicrobial agents compared to defense peptides produced by Paneth cells. However, they should not be underestimated as potential alternatives to classic antibiotics, given their potent effects as neuroimmune modulators.

An important reason to further investigate the role of gut neuropeptides on intestinal homeostasis, possibility via an effect on gut microbiota composition, has to do with the fact that several neuropsychiatric disorders, which are associated with dysbiosis in the gut and intestinal inflammation, have also been described to be associated with altered levels of (gut) neuropeptides, though it is not yet clear whether disrupted levels of these neuropeptides are also detected in the GIT. For example, Autism spectrum disorder (ASD), which is characterized by a wide range of symptoms, including immune dysregulation, gut microbiota dysbiosis, and gastrointestinal dysfunction (Vuong and Hsiao 2018), has been linked with increased circulating levels of CGRP (Nelson et al. 2001). Major depressive disorder (MDD) seems to also be associated with increased levels of SP, which are restored after antidepressant treatment (Bondy et al. 2003; Lieb et al. 2004), together with altered levels of NPY (Morales-Medina et al. 2010). Another example is Parkinson’s disease (PD), a neurodegenerative disorder that presents gastrointestinal dysfunction and dysbiosis as comorbidities (Poirier et al. 2016; Sun and Shen 2018). Although more clinical trials are required to obtain consistent results, SP, NPY, and CGRP levels also seem to be altered in PD patients (Svenningsson et al. 2017; Thornton and Vink 2008). Finally, it is worth mentioning that a direct influence of gut microbiota on eating disorders has also been shown in the last years. Increased levels of bacterial induced α-MSH autoantibodies have been reported in patients with anorexia nervosa, bulimia, and binge eating disorder. The same study found that induction of α-MSH autoantibodies in mice was able to influence food intake, anxiety, and melanocortin signaling (Tennoune et al. 2014).

Taken together, there is sufficient data from both animal and human models that point to a key role of gut microbiota in neuropsychiatric disorders, which might be mediated in part by gut neuropeptides and their direct and indirect functions on the gut microbiota itself and the brain. However, more insights into the mechanisms are needed before any solid conclusion can be made. For example, there is no evidence regarding whether the elevated levels of these neuropeptides have an origin in the gut or how altered levels of circulating neuropeptides correlate with the progressing of intestinal inflammation in these disorders. Given the strong association of intestinal microbial dysbiosis with many neuropsychiatric or neurodegenerative disorders, it is worth addressing these questions which could potentially reveal gut neuropeptides as promising therapeutic agents in these pathological conditions.
